# Choosing Wisely

**DOI:** 10.1007/s11606-025-10001-1

**Published:** 2025-11-19

**Authors:** Sarah Hinton

**Affiliations:** Departments of Pediatrics and Internal Medicine, Prisma Health/University of South Carolina School of Medicine Greenville, Greenville, SC USA

**Keywords:** academic medicine, faculty development, narrative medicine

The email pinged, and I noticed the subject line: “Department of Medicine Awards.” This was different from the usual calendar invitation I receive as the Med-Peds residency program director, which merely reminds me of the date and time so I can celebrate my residents and colleagues. This email signaled that I was the recipient of an award. I was utterly shocked and deeply appreciative to receive this honor—my first award in medicine. Throughout my medical training, I had often found myself somewhere in the middle—not an all-star, but not a problem learner either. As an early-career physician in academic medicine, my focus was on doing good work for my patients and learners, and the idea of being recognized for my clinical work or teaching genuinely meant a lot to me on a personal level. With anticipation, I opened my calendar to mark the date, only to have my heart sink. The Department of Medicine Awards Ceremony was scheduled for the same morning as Muffins with Mom at my son’s school, creating a scheduling conflict. In that moment, I recalled the wise words of one of my mentors about the importance of attending my children’s events: “They may not remember when you were there, but they will always remember when you weren’t.”

Always the optimist and determined to “make things work,” I thought I could attend the beginning of Muffins with Mom and leave early, arriving just 15 min late to the awards ceremony, which should still allow me enough time to accept my award. This was going to work. It had to.

I woke up early to prepare for my big morning. However, my youngest woke up on the wrong side of the bed, cried all morning, and dillydallied while getting dressed. I quickly passed her off to my husband to take her to daycare while my son and I piled into the car to head to school. We were only running a few minutes late. I figured I could still make it work.

When I arrived at school, I was faced with a long line of cars. A feeling of dread washed over me as I realized that most other families had arrived on time and that my carefully planned morning was falling apart. I eventually found a parking spot and hurriedly dragged my son into the school. As we approached the cafeteria for Muffins with Mom, my heart nearly stopped. The line extended all the way out of the cafeteria and down the hallway. I was already behind schedule and needed to leave in 5 minutes. Panic started to set in.

I smiled at my son as he excitedly pointed out his friends while the line inched forward. However, I felt anxiety brewing inside me as I silently wished the line would move faster. By the time we managed to sit down with our box-store-bought muffins and orange juice, it was already 5 minutes past my planned departure time. My son caught me glancing at the clock and looked down, clearly disheartened. “It’s okay, Mom. You can go. I know you need to get to work,” he said, forcing a look of reassurance. In that moment, I realized I had ruined the magic of what this experience was supposed to be. I tried to salvage the moment with a heartfelt hug, but my son’s disappointment was palpable as he walked away to his classroom. As I walked away from his school, I felt disappointment in myself for rushing through something that was meant to be special for both of us; yet, I still held onto the hope of enjoying my moment of accepting my first professional award since starting medical training.

At this point, I was well behind schedule and would miss half of the awards ceremony. I texted a colleague with my projected ETA, and she responded, “Hurry.” I logged onto the ceremony virtually and tried to type “Congratulations!” at red lights after each award was announced. With only a quarter of a mile to go, my colleague texted, “Are you close?” “Almost,” I replied. Minutes later, as I pulled into the parking lot, I heard the department chair say, “Next, we will give out our awards for Outstanding Primary Care Educator.” My heart sank, and I felt close to tears, knowing I would miss my moment of recognition. I heard him call my name and read a beautiful paragraph from my nomination just as I hurried toward the conference room. By the time I entered the room, they had already moved on to the next award. I sat in the back, silently holding back tears of frustration and disappointment. In my attempt to attend two events where I was being honored, I ended up missing out on both experiences. Instead of being celebrated, I felt defeated. Any working parent can tell you that there are days when you might excel as a parent but fall short professionally and vice versa. On this day, I aimed to hit the mark in both but ended up failing altogether.

I wish I could say that this was the last time something like this happened. However, I often find myself navigating the challenging crossroads of being a mother and a doctor. I recently found a LinkedIn article that offers a thoughtful framework for handling these situations.^1^ The recommendations in the article are as follows: (1) Assess the urgency of each obligation, (2) evaluate the importance of the occasion, (3) identify opportunities to be flexible with deadlines or obligations, (4) set and communicate clear boundaries, and (5) prioritize health. In addition to the article’s wise advice, I have discovered for myself that the most helpful strategy is embracing the reality that there will inevitably be times when I have to choose between personal and professional obligations. When caught in these scenarios, I aim to choose wisely and to be fully present in whatever experience I choose.
